# Diversity of Virulence Factors Associated with West Australian Methicillin-Sensitive* Staphylococcus aureus* Isolates of Human Origin

**DOI:** 10.1155/2016/8651918

**Published:** 2016-05-09

**Authors:** Charlene Babra Waryah, Jully Gogoi-Tiwari, Kelsi Wells, Karina Yui Eto, Elnaz Masoumi, Paul Costantino, Michael Kotiw, Trilochan Mukkur

**Affiliations:** ^1^School of Biomedical Sciences, Faculty of Health Science, Curtin Health Innovation Research Institute, Curtin University, Bentley Campus, Perth, WA 6102, Australia; ^2^School of Health, Nursing and Midwifery, University of Southern Queensland, Toowoomba, QLD 4350, Australia

## Abstract

An extensive array of virulence factors associated with* S. aureus* has contributed significantly to its success as a major nosocomial pathogen in hospitals and community causing variety of infections in affected patients. Virulence factors include immune evading capsular polysaccharides, poly-N-acetyl glucosamine, and teichoic acid in addition to damaging toxins including hemolytic toxins, enterotoxins, cytotoxins, exfoliative toxin, and microbial surface components recognizing adhesive matrix molecules (MSCRAMM). In this investigation, 31 West Australian* S. aureus* isolates of human origin and 6 controls were analyzed for relative distribution of virulence-associated genes using PCR and/or an immunoassay kit and MSCRAMM by PCR-based typing. Genes encoding MSCRAMM, namely, Spa, ClfA, ClfB, SdrE, SdrD, IsdA, and IsdB, were detected in >90% of isolates. Gene encoding *α*-toxin was detected in >90% of isolates whereas genes encoding *β*-toxin and SEG were detectable in 50–60% of isolates. Genes encoding toxin proteins, namely, SEA, SEB, SEC, SED, SEE, SEH, SEI, SEJ, TSST, PVL, ETA, and ETB, were detectable in >50% of isolates. Use of RAPD-PCR for determining the virulence factor-based genetic relatedness among the isolates revealed five cluster groups confirming genetic diversity among the MSSA isolates, with the greatest majority of the clinical* S. aureus* (84%) isolates clustering in group IIIa.

## 1. Introduction

*Staphylococcus aureus* is a frequent opportunistic pathogen known to cause a wide variety of diseases ranging from skin infections such as boils and carbuncles to more serious infections such as toxic shock syndrome, endocarditis, pneumonia, and sepsis [1–4]. This has led to the emergence of* S. aureus* as a common cause of hospital acquired and community acquired infections [5, 6].

The pathogenesis of* S. aureus* is attributed to several virulence factors including biofilm formation and associated prolonged persistence of antibiotic resistance and the production of a wide array of toxins [5, 7]. A biofilm or slime, defined as a congregation of microorganisms residing in a protective extracellular matrix [8, 9], constitutes the first step in initial attachment followed by colonization and subsequent infection. Colonization is commonly associated with an assortment of adherence factors or adhesins which aid bacterial attachment to the host surface using microbial surface component recognizing adhesive matrix molecules (MSCRAMM). Over 20 different MSCRAMM, which can be expressed in* S. aureus*, have been identified [10]. Major protein adhesins in this group include biofilm-associated protein (Bap), clumping factors A and B (ClfA, ClfB), fibronectin binding proteins A and B (FnBPA, FnBPB), collagen binding protein (Cna), bone sialoprotein binding protein (Bbp), iron regulated surface determinants A and B (IsdA, IsdB), serine aspartate repeat gene proteins D and E (SdrD, SdrE), and Protein A (Spa) [11–14]. Following adherence, the biofilm is further strengthened by an intracellular adhesin encoded by the* ica* operons (*ica*A,* ica*B,* ica*C, and* ica*D genes) which produce the cell surface polysaccharide poly-N-acetyl *β*-1-6 glucosamine (PNAG) and another antigen 336, a derivative of cell wall teichoic acid [13, 15, 16]. A strong relationship between PNAG and biofilm formation, although not absolute, was previously reported [8, 17].

In addition to the possession of MSCRAMM,* S. aureus* also produces a range of exotoxins that aid in host tissue membrane disruption providing nutrients essential for bacterial cell growth [18, 19] with some also contributing to biofilm formation. Exotoxins produced include cytotoxins, Panton Valentine Leucocidin (PVL), and hemolysins (*α*, *β*, and *γ*), which possess the ability to form pores in host cells enabling lysis [20, 21]. Additional toxins encoded for and/or produced include toxic shock syndrome toxin (TSST-1) and the staphylococcal enterotoxins or SE (SEA-SEE, SEG-SEJ), some of which are best characterized as superantigens in reference to their ability to activate the proliferation of T-cells leading to release of increasing levels of proinflammatory cytokines [22, 23]. These also include the rare and virulent exfoliative toxins ETA and ETB [24].

The increasing trend towards development of persistent antibiotic resistance improves the ability of this pathogen to resist treatment with antibiotics [5, 25] a fundamental feature in the development of chronic infections. Aim of this study was to determine the diversity of distribution of the major MSCRAMM and toxins among the West Australian* S. aureus* isolates of human origin, using serological and/or genotypic analysis and determine their genetic relatedness.

## 2. Materials and Methods

### 2.1. Collection of Strains

A total of 19 human* S. aureus* strains donated by different clinical pathology laboratories to the School of Biomedical Sciences in West Australia were kindly donated by Mr. Alain Delhaize, Senior Technical Manager, responsible for managing this collection. The remaining 12* S. aureus* isolates were collected from the laboratory medicine students enrolled in medical microbiology (Human Ethics approval number SoBS 04/11) and 5 accredited capsular (CP) positive or negative control strains were kindly provided by Professor Gerald Pier, Channing Laboratory, Brigham and Women's Hospital. The 5 accredited CP positive or negative control strains used in this investigation included Strain M (CP1), Smith Diffuse (CP2), Strain Newman (CP5), USA 400 (CP8), and LAC USA 300 (CP neg). The 6th control strain was ATCC® 29213*™*, a strong biofilm former. All strains were subjected to preliminary microbiological testing to confirm* S. aureus* [26] and methicillin-sensitivity (MSSA) as described elsewhere [5]. All* S. aureus* strains were stored at −80°C on cryobeads (Blackaby Diagnostic Pty Ltd., WA) for future studies. Positive ATCC toxin typing controls used in this study were ATCC 13565*™* for *β*-hemolysin, ATCC 49775*™* for PVL and *γ*-hemolysin, ATCC 51651*™* for TSST-1, and ATCC 8096*™* for *α*-hemolysin.

### 2.2. Bacterial Strain Growth

Pure colonies of* S. aureus* strains were inoculated in sterile nutrient broth dispensed in McCartney vials and incubated at 37°C for 24 hrs in a shaker incubator.

### 2.3. DNA Extraction

All strains were subjected to DNA extraction using the Mo-Bio DNA Extraction Kit (MO BIO Laboratories, Inc., Carlsbad, CA). All extracts were stored at −20°C until used.

### 2.4. Detection of Genes Encoding PVL and mecA

Utilization of the GenoType® MRSA assay (Hain Lifesciences) was used for detection of PVL and the presence of methicillin resistance. Briefly, DNA was isolated from cultured media and amplified with biotinylated primers. The amplified product was bound using a DNA strip technology that permitted visual identification of the presence of* mecA* and PVL genes in* S. aureus*.

### 2.5. Detection of* S. aureus* Enterotoxins

A SET-RPLA Toxin Detection Kit purchased from Thermo Fisher Scientific Australia was used to serologically type SEA, SEB, SEC, and SED. Briefly, latex sensitized with a combination of antienterotoxin A–D types serially diluted and added to the bacterial suspension. After 24 hrs incubation at room temperature, each well was observed for agglutination, which indicated the presence of enterotoxins.

### 2.6. Genotyping of* S. aureus* Strains

Determination of the presence of enterotoxins, mentioned in Section 2.5, was further confirmed by genotyping. Because the scope of detection of the exotoxins produced by the* S. aureus* isolates was limited because of the lack of availability of serological kits, the presence of a number of other toxins, described below, was carried out by genotyping.

The primers used in this investigation with their respective melting temperature (*T*_*m*_), band size, and references are shown in Table 1. Briefly, the conditions used for detection of different virulence factors were as follows.

Amplification of* TSST-1*,* clfA*,* clfB*,* can,* and* spa* was performed at 95°C for 5 min, 30 cycles of 95°C for 30 sec, *T*_*m*_ for 30 sec, and 72°C for 45 sec with a final extension of 72°C for 10 min.

Amplification of* fnBpA*,* fnBpB*,* hlb*,* sdrE*,* bbp*,* isdA,* and* sdrD* and* sdrE* genes was performed at 95°C for 5 min, 35 cycles of 95°C for 30 sec, *T*_*m*_ for 30 sec, and 72°C for 45 sec with a final extension of 72°C for 10 min. Primers for* isdB* were developed in this study and amplified with the following conditions at 35 cycles of 95°C for 30 sec, *T*_*m*_ for 1 min, and 72°C for 2 min with a final extension of 72°C for 10 min.

Amplification of* hla* genes was performed at 95°C for 5 min, 38 cycles of 95°C for 30 sec, *T*_*m*_ for 30 sec, and 72°C for 45 sec with a final extension of 72°C for 10 min. While amplification of* sea*,* seb*,* sec*,* sed*,* see*,* seg*,* seh*,* sei,* and* sej* was performed at 95°C for 5 min, 30 cycles of 95°C for 2 min, *T*_*m*_ for 1 min, and 72°C for 1 min with a final extension of 72°C for 5 min, amplification of* eta* and* etb* was performed at 95°C for 5 min, 30 cycles of 95°C for 1 min, 58°C for 1 min, and 72°C for 1 min with a final extension of 72°C for 10 min. Amplification of* hlb* was performed at 95°C for 5 min, 35 cycles of 95°C for 45 sec, *T*_*m*_ for 45 sec, and 72°C for 1 min with a final extension at 72°C for 10 min.

All PCR products were subjected to electrophoresis on a 1.5% agarose gel and stained with 0.8 *μ*L/100 mL of Midori Green DNA Stain (Nippon Genetics) in a 1x Sodium Borate Buffer (1x SB Buffer). O'RangeRuler DNA Ladder, 100–1500 bp (Fermentas), was used to observe approximate band sizes on the gel which was visualised on UV transilluminator.

### 2.7. RAPD Analysis

Three sequence primers previously published were used for RAPD-PCR test to provide more information on clinical, student, and control strains used in this study [36]. Primers C (5′-AGGGAACGAG-3′), OPA9 (5′-GGGTAACGCC-3′), and OPA13 (5′-CAGCACCCAC-3′) were used for amplification using 1 cycle of 94°C for 60 sec, 35 cycles of 94°C for 35 sec, 33°C for 30 s, and 72°C for 65 sec, followed by 1 cycle of 72°C for 7 min [36].

All PCR products were run on a 1% agarose gel in 1x SB Buffer. Gel was stained with Midori Green and viewed under UV transilluminator. Bacterial DNA was randomly selected to run in duplicate to ensure reproducibility of amplification. Bands were scored in binary code with a factor of 1 representing presence of band and a factor of 0 representing absence of bands. Results of the 3 primer sets were banded to produce a dendrogram using UPMA (DenoUPMA, http://genomes.urv.cat/UPGMA/index.php) and using the Jaccard coefficient to determine the relatedness and level of similarity between the isolates used in this study.

## 3. Results and Discussion

Several MSCRAMM were detected by genotyping in a high percentage of* S. aureus* isolates. These included genes encoding the proteins ClfA, ClfB, Spa, SdrD, SdrE, IsdA, and IsdB (Table 2). On the other hand, genes encoding the Bbp, FnBpB, and Cna proteins were detectable in less than 50% of the isolates, gene encoding FnBpA protein being detectable in the smallest percentage of the isolates.

The average number of MSCRAMM detected in this study was approximately 7, with 27 strains having a range of >6–10 (data not shown). In only 4/31 strains, 5 MSCRAMM or less were detected. Compiled results for MSCRAMM typing are shown in Table 2.

Among the toxins, the most prevalent toxin detected by genotyping among the* S. aureus* isolates was *α*-toxin, 2nd and 3rd most prevalent detected toxins being the enterotoxin G and *β*-toxin (Table 3). The genes encoding other toxins were prevalent in less than 30% of the isolates, with the lowest ones being the exfoliative toxins A and B. No strain was positive for genes encoding PVL toxin.

Twenty-three strains possessed genes encoding 2–4 different types of toxins. Only 3 strains possessed the gene for one toxin and 5 strains expressed genes for >5 toxins. The average number of toxins produced by the* S. aureus* strains in this study was 3 toxins (data not shown).

The SET-RPLA Toxin Detection Kits were able to detect fewer toxins as compared to SE genotyping (Table 4). Of the 8 SEA positive* S. aureus* strains, only 3 were detected in serotyping and, of 6 SEB positive strains, only 1 was detected in serotyping (Table 4). Of the 3 SEC positive strains, only 2 were detected by serotyping; however the genotyping and serotyping correlated with 0 positives by both methods (not significant at *p* < 0.05 level but substantial at *p* < 0.06).

PCR typing was more sensitive than immunoassays in detecting the genes associated with toxin production.

Accredited test capsular control strains were not positive for genes encoding SED, SEE, PVL, ETA, or ETB. All test control strains were positive for *α* and *β* and the TSST toxins and Spa, ClfA, ClfB, SdrE, and SdrD MSCRAMM (Table 5).

Smith Diffuse* S. aureus* (CP2) expressed 9 MSCRAMM and 9 toxins, the highest of the control strains. Strain M (CP1) expressed 9 MSCRAMM and 8 toxins, USA 400 MW2 (CP8) expressed 9 MSCRAMM and 7 toxins, LAC USA 300 (CP neg) expressed 9 MSCRAMM and 6 toxins, ATCC 29213 expressed 8 MSCRAMM and 7 toxins, and Strain Newman (CP5) expressed 8 MSCRAMM and 6 toxins.

**Figure 1:**
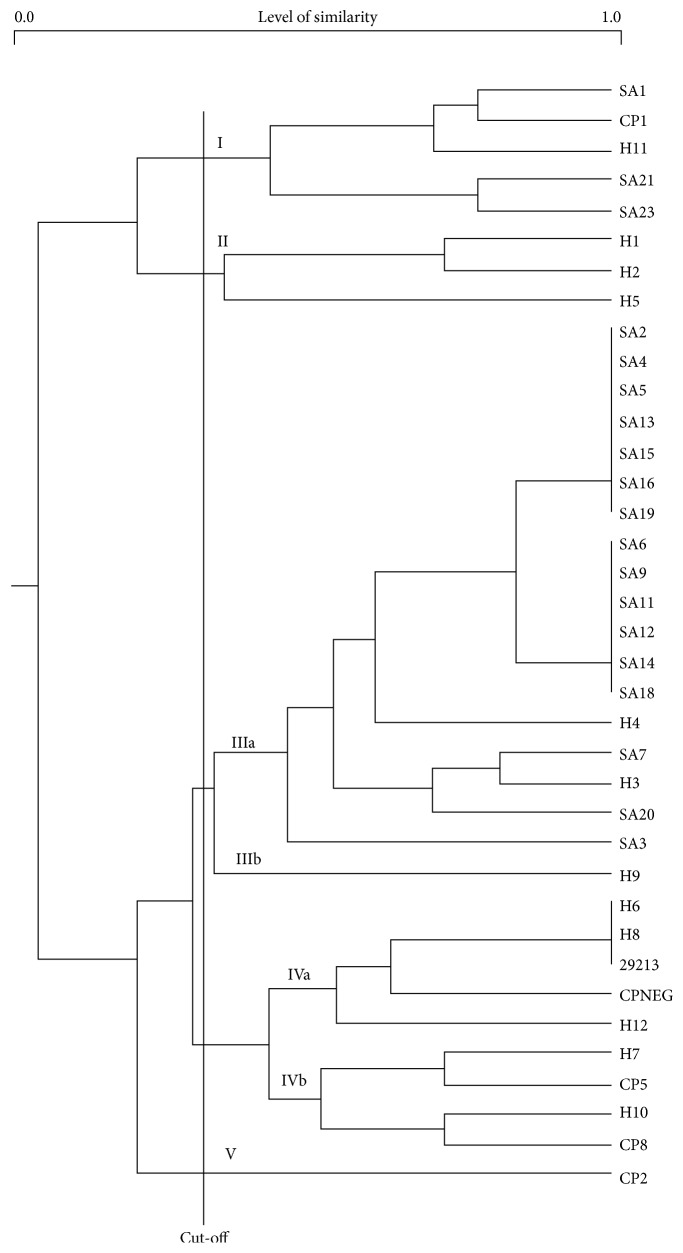
RAPD-based dendrogram indicating the genetic relatedness among* S. aureus* isolates including the control isolates.

Amplification with primers OPA09 and OPA13 yielded 4 RAPD patterns from 3 distinct bands each whereas amplification with primer C yielded 6 RAPD patterns from 4 distinct bands. Presence or absence of bands resulted in binary data that was analyzed to produce a dendrogram. Using RAPD analysis, 5 cluster groups displaying the distribution of MSCRAMM and toxins between the groups were discernible (Figure 1).

The cluster cut-off point was determined at 33% level of similarity (0.333) resulting in 5 major cluster groups (Table 6), namely, Cluster of Ia and Ib (level of similarity 0.667 to 0.800), Cluster of IIa and IIb (level of similarity 0.333 to 0.750), Cluster of IIIa and IIIb (level of similarity 0.333 to 1.000), Cluster of IVa and IVb (level of similarity 0.500–0.600), and Cluster of V (level of similarity 1.000), which were used to compare the cluster groups (Table 6).

It can be seen that the majority of* S. aureus* isolates were clustered into group IIIa, with 58% (18/31) of the isolates displaying clonal similarity of MSCRAMM and toxins. The majority of clinical strains (16/19) were clustered in group IIIa suggesting a common source of infection of patients in hospitals (Table 7). Student strains, on the other hand, were dispersed into several cluster groups (Ia, IIa, IIb, IIIa, IIIb, Iva, and IVb) indicating multiple potential sources for acquisition of infection. The control strains were clustered in groups Ia, IVa, and IVb.

Greater diversity of cluster groups associated with the student MSSA isolates also indicated greater diversity of their clonal origins.

MRSA strains are known to be highly clonal [37]. However, unlike the diversity reported recently with MSSA isolates in Europe, genetic diversity of virulence factors associated with clonal complexes of West Australian MSSA isolates has not yet been reported. Vandendriessche et al. [38] demonstrated high genetic diversity of MSSA carriage isolates from animals and humans on pig, veal, dairy, beef, and broiler farms using* spa* typing and multilocus sequence typing (MLST). These studies supported a previous report [39] on the heterogeneity and genetic diversity of MSSA isolated from clinical specimens in a teaching hospital in Germany using spa typing, MLST, and enterotoxin genotyping. In contrast, the genetic diversity of the MSSA isolates determined in this investigation was determined based on the prevalence of both MSCRAMM and enterotoxin genotyping.

A large array of virulence factors [12, 15, 19] is involved in the pathogenesis of infections caused by* S. aureus*. Whether the information gained in this study on the relative prevalence of the genes encoding different virulence antigens and confinement of the majority of WA clinical isolates to a single cluster group (IIIa) offers an opportunity for formulation of potential strategies for the development of an effective vaccine capable of combatting infections caused by this pathogen in Western Australia remains to be determined.

Strategies used for the development of vaccines against infections caused by* S. aureus* targeting a limited number of single antigens is unlikely to be effective for global vaccine usage because of differences in the distribution of genes encoding different virulence factors participating in the establishment of infection. Of interest is a relatively recent study in which patients afflicted with* S. aureus* bacteremia were reported to display different antibody responses to 19 different MSCRAMM of each bacterial strain that was isolated from these patients [25]. Ideally, an effective* S. aureus* vaccine must generate protective immunity that can neutralize the major exotoxins and interfere with adhesion facilitated by the major MSCRAMM participating in attachment/colonization of this pathogen to the niche host tissue. Many different types of vaccines including MSCRAMM-based vaccines [40], capsular polysaccharide, and/or PNAG-based conjugate vaccines [3, 15, 40] involving conjugation of one to 3 MSCRAMM [27, 40] or selected inactivated toxins including *α*-toxin encoded by the* hla* gene [3, 28, 40–43] have been evaluated using passive and/or active immunization of mice. However, none of these vaccines were considered to be providing satisfactory protection raising doubts on the possibility of ever developing an effective vaccine against* S. aureus* infections for use in humans [44], particularly after the report of antigenic competition subsequent to coadministration of CP-based and PNAG-based conjugate vaccines [45]. Fortunately, not all the potential options for the development of an effective vaccine against infections caused by* S. aureus* have been exhausted if one was to take the relative distribution/prevalence and cluster grouping of virulence antigens among the clinical isolates into account for the development of a universal vaccine against infections caused by* S. aureus*.

## Figures and Tables

**Table 1:** Primers used for detection of exotoxins and MSCRAMM using conventional PCR.

Proteins targeted	Primer forward (5′-3′)	Primer reverse (5′-3′)	*T*_*m*_	Expected band size (bp)	Reference
Cna Collagen binding protein	AAA GCG TTG CCT AGT GGA GA	AGT GCC TTC CCA AAC CTT TT	50°C	192	[1]
ClfA Clumping factor A	CGC CGG TAA CTG GTG AAG CT	TGC TCT CAT TCT AGG CGC ACT T	55°C	314	[27]
ClfB Clumping factor B	ATG ATC TTG CTT GCG TT	CCG ATT CAA GAG TTA CAC C	47°C	215	[27]
Spa Protein A	TCA AGC ACC AAA AGA GGA AGA	GTT TAA CGA CAT GTA CTC CGT TG	51°C	Variable	[28]
FnBPA Fibronectin binding protein A	GCG GAG ATC AAA GAC AA	CCA TCT ATA GCT GTG TGG	48°C	1279	[29]
FnBPB Fibronectin binding protein B	GGA GAA GGA ATT AAG GCG	GCC GTC GCC TTG AGC GT	56°C	820	[29]
Bbp Bone sialoprotein binding protein	AAC TAC ATC TAG TAC TCA ACA ACA G	ATG TGC TTG AAT AAC ACC ATC ATC T	53°C	575	[30]
IsdA Iron regulated surface determinant A	CTG CGT CAG CTA ATG TAG GA	TGG CTC TTC AGA GAA GTC AC	52°C	332	[25]
IsdB Iron regulated surface determinant B	ACG AGA GTT TGG TGC GCT AT	GTT GAG GCC CCT ACT TCT GA	55°C	192	This study
SdrD Serine aspartate repeat gene D	CGG AGC TGG TCA AGA AGT AT	TGC CAT CTG CGT CTG TTG TA	52.3°C	500	[25]
SdrE Serine aspartate repeat gene E	AGA AAG TAT ACT GTA GGA ACT G	GAT GGT TTT GTA GTT ACA TCG T	50°C	433	[31]
TSST-1 Toxic shock syndrome toxin	ACC CCT GTT CCC TTA TCA TC	TTT TCA GTA TTT GTA ACG CC	53°C	326	[32]
ETA Exfoliative toxin A	GCA GGT GTT GAT TTA GCA TT	AGA TGT CCC TAT TTT TGC TG	58°C	93	[33]
ETB Exfoliative toxin B	ACA AGC AAA AGA ATA CAG CG	GTT TTT GGC TGC TTC TCT TG	58°C	226	[33]
Hla Alpha toxin	GTA CTA CAG ATA TTG GAA GC	GTA ATC AGA TAT TTG AGC TAC	47°C	274	[34]
Hlb Beta toxin	GCC AAA GCC GAA TCT AAG	CGC ATA TAC ATC CCA TGG C	51°C	840	[29]
SEA Staphylococcal enterotoxin A	TTG GAA ACG GTT AAA ACG AA	GAA CCT TCC CAT CAA AAA CA	50°C	120	[35]
SEB Staphylococcal enterotoxin B	TCG CAT CAA ACT GAC AAA CG	GCA GGT ACT CTA TAA GTG CC	50°C	478	[35]
SEC Staphylococcal enterotoxin C	GAC ATA AAA GCT AGG AAT TT	AAA TCG GAT TAA CAT TATA CC	50°C	257	[35]
SED Staphylococcal enterotoxin D	CTA GTT TGG TAA TAT CTC CT	TAA TGC TAT ATC TTA TAG GG	50°C	317	[35]
SEE Staphylococcal enterotoxin E	AGG TTT TTT CAC AGG TCA TCC	CTT TTT TTT CTT CGG TCA ATC	50°C	209	[35]
SEG Staphylococcal enterotoxin G	AAG TAG ACA TTT TTG GCG TTC C	AGA ACC ATC AAA CTC GTA TAG C	55°C	287	[35]
SEH Staphylococcal enterotoxin H	GTC TAT ATG GAG GTA CAA CAC T	GAC CTT TAC TTA TTT CGC TGT C	48.4°C	213	[35]
SEI Staphylococcal enterotoxin I	GGT GAT ATT GGT GTA GGT AAC	ATC CAT ATT CTT TGC CTT TAC CAG	50°C	454	[35]
SEJ Staphylococcal enterotoxin J	CAT CAG AAC TGT TGT TCC GCT AG	TGA ATT TTA CCA TCA AAG GTA C	50°C	142	[35]

**Table 2:** Distribution of MSCRAMM detected by genotyping.

Gene encoding	Number of positive isolates (%)
SpaA	28 (90.32%)
FnBPA	2 (6.45%)
FnBPB	13 (41.93%)
Cna	12 (38.71%)
ClfA	26 (83.87%)
ClfB	27 (87.1%)
SdrD	28 (90.32%)
SdrE	30 (96.77%)
Bbp	14 (45.16%)
IsdA	28 (90.32%)
IsdB	30 (96.77%)

**Table 3:** Distribution of different toxins detected by genotyping and/or serotyping.

Encoding gene	Number of positive isolates (%)
Staph enterotoxin A	8 (25.8%)
Staph enterotoxin B	6 (19.35%)
Staph enterotoxin C	3 (9.68%)
Staph enterotoxin D	0 (0%)
Staph enterotoxin E	0 (0%)
Staph enterotoxin G	19 (61.29%)
Staph enterotoxin H	4 (12.9%)
Staph enterotoxin I	9 (29.03%)
Staph enterotoxin J	0 (0%)
TSST-1	8 (25.8%)
PVL	0 (0%)
Alpha toxin	30 (96.77%)
Beta toxin	13 (49.93%)
Exfoliative toxin A	1 (3.23%)
Exfoliative toxin B	1 (3.23%)

**Table 4:** Correlation of serotyping versus genotyping methods for the major superantigenic enterotoxins.

Toxin	Serotyping (*n* = 31)	Genotyping (*n* = 31)	Pearson correlation coefficient *r*
SEA	3 (9.68%)	8 (25.8%)	0.553
SEB	1 (3.23%)	6 (19.35%)	0.371
SEC	2 (6.45%)	3 (9.7%)	0.891
SED	0 (0%)	0 (0%)	Not possible to calculate *r* value but it can be assumed to be 1.0

**Table 5:** Typing of control *S. aureus* strains.

Control stain	Detectable toxin genes
ATCC 29213	SEA, SEC, SEG, SEI, TSST, *α*-toxin, *β*-toxin
Strain M (CP1)	SEA, SEC, SEG, SEH, SEI, TSST, *α*-toxin, *β*-toxin
Smith Diffuse (CP2)	SEA, SEB, SEC, SEG, SEH, SEI, TSST, *α*-toxin, *β*-toxin
Strain Newman (CP5)	SEA, SEG, SEI, TSST, *α*-toxin, *β*-toxin
USA 400 MW2 (CP8)	SEA, SEC, SEG, SEH, TSST, *α*-toxin, *β*-toxin
LAC USA 300 (CP neg)	SEG, SEH, SEI, TSST, *α*-toxin, *β*-toxin

Control strain	Detectable MSCRAMM

ATCC 29213	FnBPA, Spa, ClfA, ClfB, Bbp, SdrE, SdrD, IsdA
Strain M (CP1)	FnBPA, Spa, Cna, ClfA, ClfB, SdrE, SdrD, Bbp, IsdA
Smith Diffuse (CP2)	FnBPA, FnBPB, Spa, Cna, ClfA, ClfB, SdrE, SdrD, IsdA
Strain Newman (CP5)	FnBPB, Spa, Cna, ClfA, ClfB, SdrE, SdrD, Bbp
USA 400 MW2 (CP8)	FnBPA, Spa, Cna, ClfA, ClfB, SdrE, SdrD, Bbp, IsdA
LAC USA 300 (CP neg)	FnBPA, FnBPB, Spa, Cna, ClfA, ClfB, SdrE, SdrD, Bbp

**Table 6:** Distribution of the known MSCRAMM and toxins produced by the strains used in this study.

Group	Strains and subgroups (*n*)	MSCRAMM	Toxins
I	Group Ia (3)	FnBPA, FnBPB, Spa, Cna, ClfA, ClfB, SdrE, SdrD, Bbp, IsdA, IsdB	SEA, SEC, SEG, SEH, SEI, TSST, *α*-toxin, *β*-toxin, ETA, ETB
	Group Ib (2)	FnBPB, Spa, Cna, ClfA, ClfB, SdrE, SdrD, Bbp, IsdA, IsdB	SEB, SEG, SEH, SEI, TSST, *α*-toxin, *β*-toxin

II	Group IIa (2)	FnBPa, FnBPB, SpA, ClfA, ClfB, SdrE, SdrD, IsdA, IsdB	SEB, SEG, TSST, *α*-toxin
	Group IIb (1)	FnBPB, SdrE, SdrD, IsdA, IsdB	SEC, *α*-toxin

III	Group IIIa (18)	FnBPA, FnBPb, SpA, ClfA, ClfB, Cna, Bbp, SdrE, SdrD, IsdA, IsdB	SEA, SEB, SEC, SEH, SEI, TSST, *α*-toxin, *β*-toxin
	Group IIIb (1)	Spa, Cna, ClfA, ClfB, SdrE, SdrD, IsdA, IsdB	SEC, SEG, TSST, *α*-toxin

IV	Group IVa (5)	FnBPA, FnBPB, Spa, ClfA, ClfB, SdrE, SdrD, Bbp, IsdA, IsdB	SEA, SEC, SEG, SEH, SEI, TSST, *α*-toxin, *β*-toxin
	Group IVb (4)	FnBPA, FnBPB, Cna, Spa, ClfA, ClfB, SdrE, SdrD, Bbp, IsdA, IsdB	SEA, SEC, SEG, SEH, SEI, TSST, *α*-toxin, *β*-toxin

V	Group V (1)	FnBPA, FnBPB, SpA, Cna, Clfa, Clfb, SdrE, SdrD, Bbp, IsdA, IsdB	SEA, SEB, SEC, SEG, SEH, SEI, TSST, *α*-toxin, *β*-toxin

**Table 7:** Cluster groups of clinical versus student versus control strains.

Cluster groups	Clinical strains (*n* = 19)	Student strains (*n* = 12)	Control strains (*n* = 6)
Ia	1	1	1
1b	2	0	0
IIa	0	2	0
IIb	0	1	0
IIIa	16	2	0
IIIb	0	1	0
IVa	0	3	2
IVb	0	2	2
V	0	0	1
